# Nursing care for individuals with Creutzfeldt–Jakob disease from the perspective of a nursing model and nurses: a case study

**DOI:** 10.1186/s12912-025-03476-0

**Published:** 2025-07-01

**Authors:** Ozan Acar, Aylin Özakgül, Aleyna Uçanbelen

**Affiliations:** 1 Department of Fundamentals of Nursing, Institute of Graduate Studies, İstanbul University-Cerrahpaşa, İstanbul, Türkiye; 2 Department of Nursing, Acıbadem Mehmet Ali Aydınlar University Faculty of Health Sciences, İstanbul, Türkiye; 3 Department of Fundamentals of Nursing, İstanbul University-Cerrahpaşa Florence Nightingale Faculty of Nursing, Abide-i-Hurriyet Caddesi, Şişli, İstanbul, 34381 Türkiye; 4 Department of Fundamentals of Nursing, Koç University Graduate School of Health Sciences, İstanbul, Türkiye

**Keywords:** Rare disease, Creutzfeldt–Jakob disease, Neurodegenerative disease, Nursing care, Model of living

## Abstract

**Background:**

Creutzfeldt–Jakob disease (CJD) is a progressive, contagious, and rare fatal neurodegenerative disease of the central nervous system. Its annual worldwide incidence is approximately 1–2 cases per one million, with no known nursing care and medical treatment. Thus, sharing of experiences is important.

**Aim:**

This study aimed to examine the nursing care for three patients diagnosed with CJD in line with the Model of Living and to discuss nurses’ experiences of providing care.

**Study design:**

This study used both the retrospective design and case study methods. It included three patients diagnosed with CJD who were hospitalized in the neurology ward of a university hospital in Istanbul between 2018 and 2023 and 11 nurses involved in the care of these patients. Data were collected from health records and through semistructured interviews. The health records of the patients were retrospectively analyzed and the data were systematically analyzed in line with the Model of Living. Data obtained from the face-to-face interviews with the nurses were analyzed using Malterud’s systematic text condensation method.

**Results:**

Patient care was handled specifically according to the patients’ admission and discharge status, in line with the Model of Living. From the qualitative data obtained in the case study, six main themes and eight subthemes were identified within the scope of the Strengths, Weaknesses, Opportunities and Threats analysis of nursing care experiences. The main themes were professional knowledge and experience, corporate support, lack of knowledge, emotional and physical difficulties, coping with difficulties, professional visibility, individual differences and coping with rare diseases.

**Conclusions:**

The greatest emotional difficulty of nurses was the fear of infecting/transmitting the disease.While caring for patients with CJD, nurses experienced fear of contamination and could not answer the questions of the patient’s relatives because of the uncertainty in the prognosis, potentially causing stress in them.

**Relevance to clinical practice:**

This study nurses may guide training modules or structured care recommendations on caring for patients with CJD.

**Clinical trial number:**

Not applicable.

**Supplementary Information:**

The online version contains supplementary material available at 10.1186/s12912-025-03476-0.

## What is known about the topic

- CJD has no known treatment to date, multidisciplinary palliative care is applied to manage symptoms and provide support to the individual and family for this rapidly terminal disease.
- The greatest emotional difficulty of nurses is the fear of infecting/transmitting of CJD disease.

**Supplementary Information:**

The online version contains supplementary material available at 10.1186/s12912-025-03476-0.

## What this paper adds

- Given the lack of standardized care and treatment for CJD, sharing knowledge and experience that can guide care is important.
- This study reflects to examine the nursing care provided to individuals with CJD in line with the Model of Living and to determine the experiences of nurses in caring for patients with CJD on the basis of the nurses’ perspective.
- While caring for patients with CJD, nurses experienced fear of contamination and could not answer the questions of the patient’s relatives because of the uncertainty in the prognosis, potentially causing stress in them.

## Introduction

Creutzfeldt–Jakob disease (CJD) is a progressive, contagious, and rare fatal neurodegenerative disease of the central nervous system reportedly caused by the misfolding of prion proteins in the brain [1, 2]. CJD, which is also called transmissible spongiform encephalopathies, is the most common type of human prion disease [3]. The word “prion” was derived from “*proteinaceous infectious particle*” by Stanley/İ B. Prusiner in 1982. The disease obtained its name when it was first described by the German neurologist Hans Gerhard Creutzfeldt in 1920 and soon after by Alfons Maria Jakob [4].

The annual incidence and of CJD is approximately 1–2 per one million people individuals worldwide [2]. There are approximately 350 new cases of CJD in the United States each year. One study found that the number of cases increased steadily between 2007 and 2020 in the USA, with a total of 5882 cases reported during that period, of which 3009 were female (51.2%) and 2873 were male (48.8%) [5]. Recent data have reported an increase in the incidence of sCJD, mainly due to an aging population and improved diagnostic methods [6]. Although Turkey has no national data on CJD incidence, Uslu et al. [7] reported 74 cases between 2005 and 2018. CJD has four different subtypes: sporadic, variant, iatrogenic, and familial. Sporadic CJD (sCJD) is the most common type, accounting for approximately 85% of cases [2, 8] but it has no known predisposing factor. The age group with the highest age of onset is 55–75 years. It is characterized by rapidly progressive clinical deterioration leading to death within months. Its initial symptoms could be personality and behavioral changes, including psychiatric symptoms such as hallucinations, paranoid delusions, poor memory, gait disturbance, headache, vertigo, myoclonus, extra pyramidal symptoms, and visual disturbances [3, 9]. Additionally, the initial symptoms may mimic neurological diseases such as Alzheimer’s disease, multiple sclerosis, Parkinson’s disease, encephalitis, meningitis, and acute ischemic stroke, making the diagnosis difficult, delayed, and even missed [3, 10]. As the disease progresses, akinetic mutism, myoclonus, increased startle reflex, seizure activity, decorticate or decerebrate posture, and coma may occur [9]. This rare disease has a rapidly deteriorating prognosis, with a median survival of 6 months. Death can occur in more than 90% of individuals within one year of symptom onset [1]. Although CJD has no known treatment to date, multidisciplinary palliative care is applied to manage symptoms and provide support to the individual and family for this rapidly terminal disease [9, 11].

## Background

Nurses need to provide individualized, holistic, and humanistic care for the needs of individuals diagnosed with CJD [3, 11]. In nursing care planning, knowing the nature of CJD and diagnosing patients’ responses are important so they can continue performing life activities [3, 9]. The use of a nursing model in nursing care ensures that care is provided in a holistic manner [12]. In this context, the Model of Living is frequently preferred in providing nursing care because it is suitable for contemporary nursing philosophy and is used together with the Nursing Process, a scientific problem-solving method [12]. In the 1970s, N. Roper, W. Logan and A. J. Tierney developed the Roper–Logan–Tierney Nursing Model (Model of Living), which addresses the individual in all aspects of his/her life and is frequently used in the provision of systematic nursing care [12]. The model includes activities of living (AL), lifespan, dependence/independence, and the factors influencing AL and individuality in life. The model includes 12 AL [12]. These are “maintaining a safe environment”, “communicating”, “breathing”, “eating and drinking”, “eliminating”, “personal cleansing and dressing”, “controlling body temperature control”, “mobilising”, “working and playing”, “expressing sexuality”, “sleeping” and “dying” [12].

Given the lack of standardized care and treatment for CJD, sharing knowledge and experience that can guide care is important. While several cases and case series reflecting the knowledge and experiences related to CJD have been reported, publications on nursing care remain few [10]. However, there are no studies that address CJD nursing care separately or together with a nursing model/theory and the perspective of nurses. The study’ originality was emphasized. In addition, guidelines for the management of CJD symptoms with pharmacological and nonpharmacological methods are still unavailable [11]. However, no case-based analysis was found in the nursing literature in Turkey. Accordingly, this study aimed to examine the nursing care provided to individuals with CJD in line with the Model of Living (The health records of the patients were retrospectively analyzed) and to determine the experiences of nurses in caring for patients with CJD on the basis of the nurses’ perspective (qualitative study). The questions of this study were as follows:

1. How is the nursing care for individuals with CJD in line with the Model of Living? (retrospective study)
2. What are the experiences of nurses providing care for individuals with CJD? (qualitative study).

## Methods

### Study design

This study was conducted in two stages. A retrospective research design was used for the quantitative analysis, and the case study method for the qualitative analysis. The case study method was employed because the nursing care for patients with CJD was examined in depth from the nurses’ perspective, in accordance with the nature and purpose of case studies [13].

Case study is a method in which a single situation (CJH) or event is addressed in depth with all its dimensions (nursing care patient records/nursing perspective), data are collected systematically (examining patient records/documents in line with the Life Model) and what happens in the real environment (nurse experiences).

### Population and sample

The study population consisted of only three patients because of case rarity diagnosed with CJD admitted to the neurology service of a university hospital in Istanbul between 2018 and 2023 and July and August 2023 between 20 nurses responsible for their care. The purposive sampling method was used in the first stage of the study. Accordingly, the three patients diagnosed with CJD were over the age of 18 years who received nursing care and treatment in the institution. For the nurses, the snowball sampling method was used, and they were asked (*n* = 11) whether another nurse was caring for the patients in the qualitative interviews. The inclusion criteria were as follows: being hospitalized with CJD diagnosis in the hospital where the study was conducted between 2018 and 2023 and being involved in the care of at least one of the three patients and still working in the institution.

### Data collection

In the first phase of the study, data from the health records, patients’ health history, and nursing care notes were analyzed. The data were adapted to Roper–Logan–Tierney Nursing Model (Model of Living) as early stage and progressive stage. In the second phase, we used a semistructured interview form (six question) prepared for nurses involved in the nursing care for individuals diagnosed with CJD. The interview guide was developed for this study (Suppl 1). In accordance with the form, the interviews started with collecting data on demographic profile, professional working time, and experience duration in caring for patients, continued with asking questions about care experiences in line with the Strengths, Weaknesses, Opportunities and Threats (SWOT) analysis [14, 15]. In line with the form, the time and place of the face-to-face interviews with the nurses were determined. The interviews were conducted during the nurses’ free time, such as lunch break/after work, in their rest area with a quiet, stimulus-reduced environment, taking into account that the patients’ care should not be disrupted. The average interview duration is 15–20 min. Interview data were collected between July and August of 2023. The interviews were terminated as data saturation was reached.

### Ethical considerations

This study was conducted in accordance with the Declaration of Helsinki ethical approval. Ethical approval for this study was obtained from the Ethics Committee. Approval was obtained from the Koç University Clinical Research Ethics Committee (No: 2023.225.IRB3.103) and all applicable ethical standards and regulations were followed procedures. In addition, for the examination of health records, institutional permission (dated 16.06.2023, number: 776) was obtained from the Koç University Hospital where the study was conducted; data on personal information were not included. The nurses who agreed to participate provided their written informed consent through the Informed Voluntary Consent Form. Participating nurses were assured that were informed about the confidentiality and anonymity of their responses. Interviews with the nurses would be recorded only with a voice recorder and would be kept in accordance with the data retention principles. In data transcription, the participants were coded as Nurse 1 (N1), Nurse 2 (N2)… Nurse 11 (N11), paying attention to the principle of confidentiality.

### Data analysis

The data recorded on the voice recorder during the interviews were transcribed verbatim by one researcher. To check the transcribed text, two other researchers listened to the voice recording twice. While the creation of interview questions and determination of main themes were done with SWOT analysis. And then the reliability of the transcribed interview data was analyzed using Malterud’s systematic text condensation method [16]. In this direction, two researchers individually formed the main themes from the nurse care experiences (total impression-chaos). The main theme/subthemes were then grouped. For ensuring validity, three randomly selected nurses (N2, N4, N7) were asked for their opinions regarding the table containing the first analyses obtained from the raw data (main theme/sub-theme/participant opinions). They stated that the raw data in the table were grouped correctly, taking into account their own opinions in real life (clinical practice experiences), and that the data were satisfactory. After ensuring the validity and reliability of the data, we employed the COREQ checklist [17], which is often used in qualitative research to report the study in a comprehensive and transparent manner.

## Results

In the first phase of the study, nursing care was examined in line with the elements of the Model of Living (Table 1). It was determined that the dependencies of the patients, one of whom was in adulthood and the other two in old age, increased rapidly, starting at an early stage due to factors affecting their life activities.

**Table 1:** Nursing care for patients with Creutzfeldt–Jakob in line with the model of living (*N* = 3)

			1. Patient		2. Patient		3. Patient	
Health History			Admitted immediately to the intensive care unit with dizziness, gait and speech impairment, and hallucinations. The patient was unconscious, with no functional response. Though without previous history of falls, he was immobilized and provided with in-bed position support. Married, HT, DM, no family history. Diagnosed with CJD in 2021.		Admitted immediately to the intensive care unit with complaints of deterioration in vision and gait. The patient was unconscious, with no peripheral pull response to painful stimuli. Though without previous history of falls, she was immobile and was provided with in-bed position support. Married, DM, no pedigree. Diagnosed with CJD in 2021.		Admitted to the ward with increased complaints of unsteadiness, weakness, gait disturbance, and aggression within 4–5 months. The patient was disoriented; hence, he was followed up under restriction as a state of necessity because of conditions such as medical device withdrawal, confusion, instability, weakness, and aggression. He was immobile, with in-bed position support. Married, no family history. Diagnosed with CJD in 2022.	
Diagnostic Tests			EEG, CF, MRI		EEG, CF, MRI		EEG, CF, MRI	
Treatment Procedure			Antiepileptic drugs (Levetiracetam, Carbamazepine, Clonazepam), symptomatic treatment (in case of necessity)		Antiepileptic drugs (Levetiracetam, Carbamazepine, Clonazepam), symptomatic treatment (in case of necessity)		Antiepileptic drugs (Levetiracetam, Carbamazepine), symptomatic treatment (in case of necessity)	
**Prognosis**			**Duration of Treatment and Care**: 2 years, 4 months.2023: ex.		**Duration of Treatment and Care**: 2 years, 3 months. August 2023: transfer to external institution care/treatment ongoing		**Duration of Treatment and Care**: 2 months. October 2022: transfer to external institution care/treatment ongoing	
Lifespan			Adulthood (69 years-male)		Adulthood (50 years-female)		Adulthood (60 years-male)	
Activities of Living	Maintaining a Safe Environment		**Early stage**: Blurred consciousness		**Early stage**: Unconscious		**Early stage**: Confused, agitated	
			**Advanced stage**: Unconscious, GCS: 5		**Advanced stage**: Unconscious, GCS: 4		**Advanced stage**: Confused, agitated, GCS: 7	
	Communicating		**Early stage**: Limited response available		**Early stage**: Noncooperative		**Early stage**: Noncooperative	
			**Advanced stage**: Noncooperative		**Advanced stage**: Noncooperative		**Advanced stage**: Noncooperative	
	Breathing		**Early stage**: Respiratory distress with spontaneous respiration, resulting in desaturation and intubation		**Early stage**: Tracheostomy with home ventilator (7–8 L/min), SpO_2_ at 100%, and tracheal aspiration		**Early stage**: Respiratory distress with spontaneous respiration, resulting in desaturation and intubation	
			**Advanced stage**: Tracheostomy with home ventilator (7–8 L/min), SpO_2_ at 100%, and tracheal aspiration		**Advanced stage**: Tracheostomy with home ventilator (7–8 L/min), SpO_2_ at 98%, and tracheal aspiration		**Advanced stage** Easy breaths with home ventilator (3–4 L/min), SpO_2_ at 92–95%, and tracheal aspiration	
	Eating and Drinking		**Early stage**: Soft diet and NG tube because of the risk of aspiration caused by decreased swallowing reflex		**Early stage**: No swallowing/spitting reflex. NG tube		**Early stage**: No swallowing/spitting reflex. NG tube	
			**Advanced stage**: NG tube		**Advanced stage**: NG tube		**Advanced stage**: NG tube	
	Eliminating		**Early stage**: Diaper and urinary catheter use		**Early stage**: Diaper use		**Early stage**: Diaper use	
			**Advanced stage**: Foley catheter use; mostly with diarrhea		**Advanced stage**: Foley catheter; mostly constipated		**Advanced stage**: Foley catheter and diaper use	
	Personal Cleansing and Dressing		**Early stage**: Caregiver support		**Early stage**: Caregiver support. Stage 4 pressure injury of the sacrum and left trochanter		**Early stage**: Caregiver support	
			**Advanced stage**: Bed bath/head bath (every 2 days), oral care (4 times a day). Right/left ear stage 2; stage 4 pressure injury of the sacrum and right/left trochanter		**Advanced stage**: Bed bath/Head bath (every 2 days), oral care (4 times a day). Full recovery from stage 4 pressure injury after care provision		**Advanced stage**: Bed bath/head bath (every 2 days), oral care (4 times a day). No pressure injuries	
Activities of Living	Controlling Body Temperature Control		**Early stage**: 36.5–37.0 °C (Temporal)		**Early stage**: 36.5–37.0 °C (Tympanic)		**Early stage**: 36.5–37.0 °C (Temporal)	
			**Advanced stage**: 37.0–37.8 °C (Temporal- subfebrile)		**Advanced stage**: 37.0–37.5 °C (Tympanic)		**Advanced stage**: 36.5 °C (Temporal)	
	Mobilising		**Early stage**: Unstable gait and limited mobilization resulting from weakness		**Early stage**: Unsteadiness in walking and weakness		**Early stage**: Unsteadiness in walking	
			**Advanced stage**: Immobile, with position support provided once every 2 h		**Advanced stage**: Immobile, with position support provided once every 2 h		**Advanced stage**: Immobile, with position support provided once every 2 h. Intermittent restraint caused by agitation and aggression	
	Working and Playing		**Early stage**: Retired		**Early stage**: Employed		**Early stage**: Retired	
			**Advanced stage**: Immobile		**Advanced stage**: Immobile		**Advanced stage**: Immobile	
	Expressing Sexuality		**Early stage**: Unconscious, unable to respond to questions. Married, with 2 children		**Early stage**: Menopause at 38 years old. Married, with 2 children		**Early stage**: Disoriented, agitated, and unable to respond to questions	
			**Advanced stage**: Unconscious, unable to respond to questions		**Advanced stage**: Unconscious, unable to respond to questions		**Advanced stage**: Unconscious, unable to respond to questions	
	Sleeping		**Early stage**: 1 h during the day, 7 h at night daily		**Early stage**: 8 h at night daily		**Early stage**: 9 h at night daily	
			**Advanced stage**: Unconscious		**Advanced stage**: Unconscious		**Advanced stage**: Agitated and disturbed at night	
	Dying		Patient ex (2023)		Patient is alive, Family (grief)		Patient is alive, Family (grief)	
**Dependence/** **Independence**			All LA-dependent		All LA-dependent		All LA-dependent	
**Factors influencing AL**			***Biophysiological***: 59 years old, male, unconsciousness, tracheostomy, NG, immobility, pressure injuries. ***Psychological***: unconscious. ***Environmental***: hospital environment. ***Sociocultural***: university graduate. ***Politicoeconomic***: private health insurance.		***Biophysiological***: 50 years old, female, unconsciousness, tracheostomy, NG, immobility, pressure injuries. ***Psychological***: unconsciousness. ***Environmental***: hospital environment. ***Sociocultural***: university graduate. ***Politicoeconomic***: private health insurance.		***Biophysiological***: 60 years of age, male, tracheostomy, NG, immobile, ***Psychological***: consciousness disoriented, ***Environmental***: hospital environment, ***Sociocultural***: primary school graduate, ***Politicoeconomic***: private health insurance.	
**Individuality in Life**			The patient (adult) was dependent on the entire LA, and care was provided in line with the factors influencing the LA.		The patient (adult) was dependent on the entire LA, and care was provided in line with the factors influencing the LA.		The patient (adult) was dependent on the entire LA, and care was provided in line with the factors influencing the LA.	

HT: Hipertension, DM: Diabetes Mellitus, CJD: Creutzfeldt–Jakob disease EEG: Electroencephalogram, CF: Cerebrospinal Fluid, MRI: Magnetic Resonance Imaging, GCS, Glasgow Coma Scale, SpO_2_: oxygen saturation, L/min: liter/minute, NG, Nasogastric

°C: Celsius, h: hour, LA: Life Activities; NG: Nasogastric

**Fig. 1:**
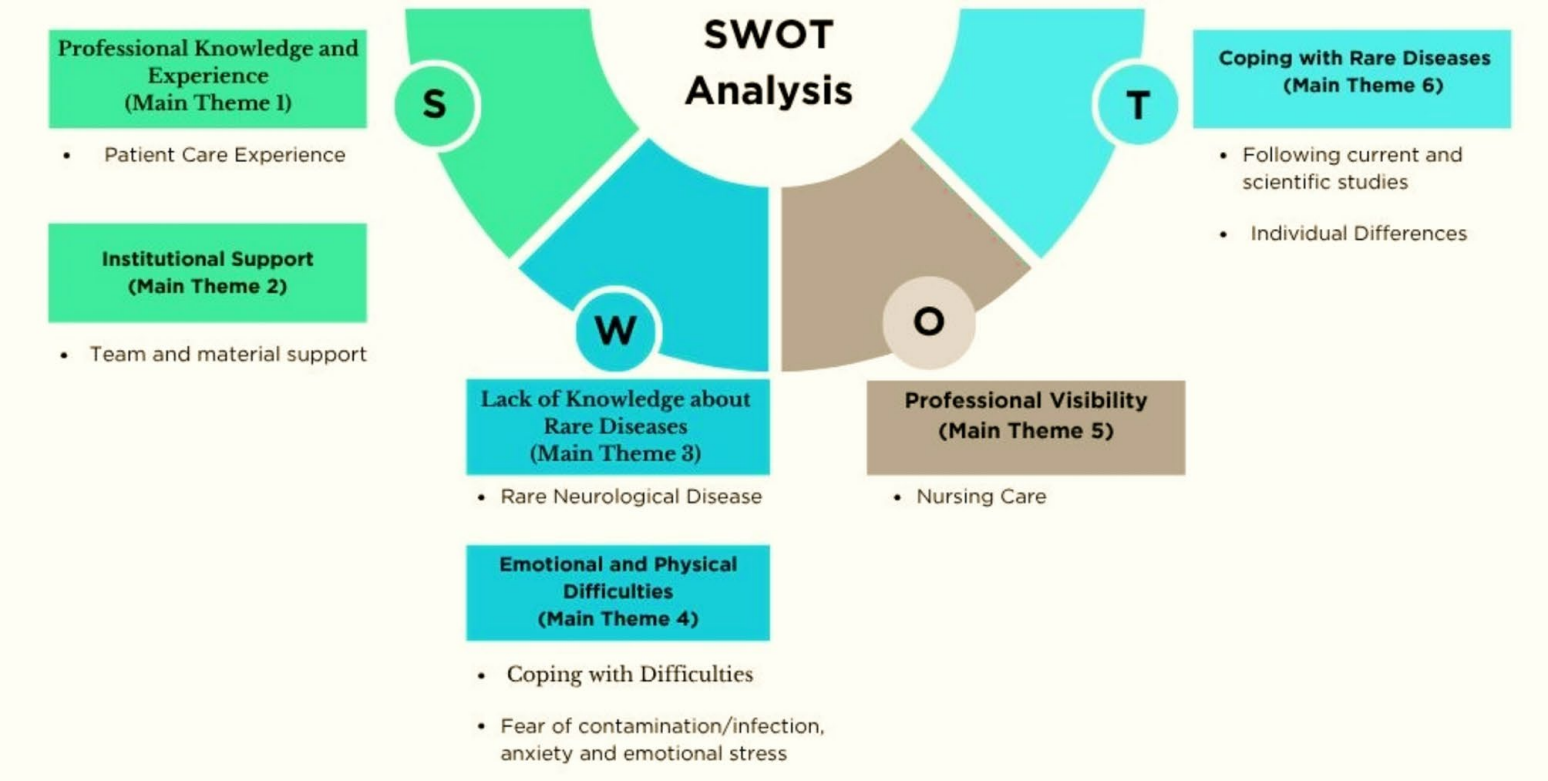
Main themes and subthemes

Nurses, except for one (H9), cared for these three patients. The duration of care they provided to patients with these three patients showed in Table 2. Six main themes and eight subthemes were identified from the qualitative interviews conducted to evaluate the nursing care for individuals with CJD from the nurses’ perspective (Fig. 1). Also, Fig. 1 showed that SWOT analysis experiences of nurses who provide nursing care. In this study, the nurses stated that their strengths in caring for individuals with CJD were that they had more professional knowledge and experience in neurology, intensive care, and palliative care (main theme 1) and that the majority (*n* = 11) had previously cared for patients with CJD hospitalized in the institution.

**Table 2:** Demographic data of nurses and the duration of care they provided to patients with CJD (*n* = 11)

Participant	Age	Work experience (month)	Neurology experience (month)	Work in the unit (month)	Study for Patient 1 (month)	Study for Patient 2 (month)	Study for Patient 3 (month)
	**Mean (Min-Max)**	**Mean (Min-Max)**	**Mean (Min-Max)**	**Mean (Min-Max)**	**Mean (Min-Max)**	**Mean (Min-Max)**	**Mean (Min-Max)**
*(N*,* nurse)*	26.00 (23–30)	47.63 (9–132)	31.00 (9–48)	28.81 (9–42)	18.18 (6–24)	18.27 (0–24)	1.81 (0–2)

- “Since I had a surgical clinic and intensive care experience before, I actually did not have much difficulty in the palliative care processes of the patients…” (Nurse 2).

Nurses also stated that institutional support (main theme 2) was one of the strengths and facilitators of care. Regarding institutional support, they indicated that support with each other as a team, personal protective equipment (PPE) support, and care material availability in the institution were important in coping with care difficulties.

- “We supported each other a lot, both psychologically and physically. They were facilitators for me…” (Nurse 10).

However, all of the nurses expressed that they were worried and anxious because CJD is a rare and unknown disease.

- “It is a very serious disease, a very rare and really incurable disease. There is no place, there is no time. This worried me a lot.” (Nurse 4).

The nurses mentioned that they did not have enough information about the transmission route of CJD and its course (uncertainty in the diagnostic process, poor prognosis) and that they did not have enough information or could not find research (main theme 3).

- “The first time I was available, I researched the transmission routes through academic research. When I looked at it, it made me feel a little more relaxed.” (Nurse 8).

Owing to the inherent infectiousness and poor prognosis of CJD, the nurses experienced emotional and physical difficulties (main theme 4). Despite knowing the routes of transmission, they were afraid of infecting themselves or their relatives because the transmission/prognosis of CJD is still poorly understood.

- “Will this harm me? Come on, I protected myself somehow, but the fear of whether I would carry it to others after leaving this room was a process that really made this process hell for me.” (Nurse 10).

Agitation or physical difficulties inherent in the nature of the disease were reflected in the nurses’ statements. They reported that they had difficulty complying with the rules of protection/isolation. In addition to emotional problems such as fear of contamination during care, all 11 nurses stated that owing to patients’ seizures and dystonia, they experienced difficulties in positioning the patients, providing hygienic care, and managing aspiration. They added that wound care was difficult.

- “We had many neurology patients, but this was the first time I had seen such a severe seizure.” (Nurse 6).

Furthermore, one nurse claimed that she had difficulty in intravenous interventions, as stated below:

- “They have a very serious peripheral disorder. You cannot easily draw blood. You cannot easily open an intravenous line. You cannot easily control blood conversion.” (Nurse 2).

Meanwhile, contamination or the fear of it led the nurses to taking certain precautions (wearing double gloves, putting scented wipes on the sides of the mask.etc.) in addition to PPE.

- “Some friends took precautions such as wearing double gloves. I prefer to wear sterile gloves because they are a little thicker…” (Nurse 2).

The nurses also mentioned that considering the patients’ poor prognosis, they experienced emotional stress while answering the questions asked by the patients’ relatives and that their empathic approach posed difficulties for them.

- “You know that you will lose the patient every day. But the reactions of the family in the process of accepting this wears us out a little bit.” (Nurse 4).

Although nurses had difficulties in caring for patients with CJD, they coped/challenge with such difficulties, owing to their strengths such as long-term patient care, interaction with the patients’ relatives, and experience (main theme 4).

- “Considering the psychology of the relatives, especially because of this situation, and with the responsibilities of our own nursing profession, I mean, this is a disease; we should think that it is no different from a patient who breaks his leg and comes here. But of course, we need to protect ourselves.” (Nurse 3).

Despite the fear of contamination, they practiced nursing care in a scientific and humanistic way.

- “It did not get ahead of it because our priority is always to be useful, of course, with the ethical principle of not harming. As a result, the person in front of me has been entrusted to me, and as I said, I think I provide the same service, the same care, and the same treatment, of course, no matter how much contact, thinking that a person’s mother and father can be my family in the same way.” (Nurse 3).

The nurses thought that successfully overcoming difficulties and reflecting the quality of care on patient outcomes were an important opportunity to make the nursing profession visible *to* patients, relatives, and other team members (main theme 5). As an indicator of effective and quality communication with patients and their relatives in care, cooperation with nurses is crucial for patients’ relatives.

- “We couldn’t leave the room for at least half an hour… This situation was noticed by the families from time to time, and they supported us in care and expressed their gratitude.” (Nurse 2).

The nurses stated that team support was present in the care. Those who participated in the interview also emphasized that the alignment of nurses’ personality traits with their professional values is important for the care of these patients. They explained that individual differences (main theme 6) could pose a threat because they may lead to differences in professional practices. At the same time, the most important threat was that nurses, especially those who lacked knowledge and experience, could be at risk of contracting infectious diseases in the process of learning care.

- “I think that nurses…should be very strong characters, I mean, for me, it is a very difficult disease for the first experience.” (Nurse 2).

For factors such as the lack of treatment for CJD, the rapidly deteriorating prognosis and death shortly after the diagnosis, the fear they experience owing to its contagious nature, and the lack or limitation of knowledge about CJD as a rare disease, the nurses recommended to follow current scientific studies that will increase their knowledge and experience without fear and anxiety for coping with rare diseases (main theme 6).

- “If they encounter this kind of phenomenon, they should, first of all, investigate the contagiousness of this disease and that it is not much different from a neurological care patient, such as encephalitis.” (Nurse 8).

## Discussion

This study examined CJD in line with a nursing model and addressed the nursing care experiences from the nurses’ perspective. The Model of Living, which is a nursing model, was used in this study because it provides an individual-specific and holistic approach while maintaining the life of a person, which is a complex phenomenon [12]. This model helps the nurses diagnose patients’ life activities according to their life span, determine the nursing diagnosis by considering the factors influencing the life activities, plan interventions according to the situation in the dependence–independence sequence when a problem arises in the activity/activities, and implement and evaluate patients’ life-specific practice and evaluation. The care that is specific to the clinical conditions of the patients in this study is similar to that of patients in previously published case series and case studies [8, 11].

In this study, the patients’ life span was in adulthood and old age, and the life span with CJD symptoms was mostly between 55 and 75 years of age, consistent with the results of the study by Harrison et al. [18] While hereditary CJD progresses very slowly, other types of this disease usually result in death within 1 year; in fact, most patients die within 6 months after symptom onset [19]. Although the CJD types of the patients in this study were not known, patient 1 received care/treatment in the same institution for 2 years and 4 months, patient 2 for 2 years and 3 months, and patient 3 for 2 months. All of these patients had longer survival times after diagnosis compared with those reported in the literature, suggesting the quality nursing care given to patients with CJD in the institution and that this nursing care was attributed to individual factors.

Currently, CJD still has no treatment to slow its progression or to ensure recovery. Therefore, nursing care is important in multidisciplinary care. When patients are diagnosed with CJD, symptom management is performed, and they are referred to the palliative care clinic, neurological clinic, or nursing home for palliative care, to improve patient comfort [11, 19]. Unlike previous studies, this study consisted of patients followed up in the clinic. The patients in the sample were dependent in all of their activities of living, implying that all interventions are required to be performed by nurses. It is stated that there is limited information on hospice care and palliative/neuropalliative care for prion diseases [18]. In this respect, sharing the experiences of nurses in these diseases is very important.

This study discusses the views of nurses regarding the nursing care they provided for patients diagnosed with CJD hospitalized in the neurology inpatient ward. Of note, the opinions of nurses in the palliative care/nursing home may differ. In terms of professional knowledge and experience, the nurse participants had a mean working time of 47.63 months and took part in the nursing care and treatment of patients with CJD in the clinic for a long time, constituting the strength of nursing care. Another strength stated by the nurses is the multidisciplinary care approach in clinical care and institutional support, such as the availability of PPE/care materials. In a study published during the Covid-19 pandemic, it was reported that there was a serious lack of personal protective equipment (PPE) in the institutions where healthcare professionals work. This shortage has led to anxiety and fear among healthcare professionals. In contrast to, the provision of PPE is reported to facilitate the patient care processes of healthcare professionals and provide an important support in this process [20]. However, given the rarity of CJD, all nurses experienced anxiety/nervousness and lacked knowledge about caring for patients with this disease. Although seizures are an unusual finding in sCJD [11], they were observed in three of the patients in this study, posing difficulties for nurses when providing care. PPE is very important in CJH, which is a contagious and long-term care disease like CJH, and should be considered different from acute care. In this study, it was important to provide PPE (corporate support) when it was requested, that is, in the long term.

Nurses are in constant contact while meeting the patient’s care needs and performing invasive procedures. Therefore, infection control is one of the most important issues [21]. Nurses experience fear because of not knowing how the disease is transmitted and not having enough information to cope with clinical problems arising from the fact that CJD is a rare disease [9]. In this study, the greatest emotional difficulty of nurses was the fear of infecting/transmitting the disease.

Some nurses, as well as those who have developed their own coping style such as taking extra precautions, thought that wearing PPE may be sufficient because CJD is an infectious disease [9]. Rapidly emerging neurological symptoms and a worsening prognosis reveal problems in care [9]. Both the affected patient and their family members also have emotional care needs [11]. In a previous study including six patients with CJD, their families did not believe in the diagnosis, and all of them experienced distress [22]. A qualitative study involving caregivers revealed the following factors: (1) the disease’s unique nature; (2) difficult diagnostic process and lack of information, as well as, clinical problems; and (3) distress as the most important care problem [18]. This previous study revealed that the most common problem experienced by caregivers of patients with CFD was the lack of clear/understandable prognostic information about clinical care [18].

While caring for patients with CJD, nurses experienced fear of contamination and could not answer the questions of the patient’s relatives because of the uncertainty in the prognosis, potentially causing stress in them. In another study investigating patients with CJD, caregivers expressed that they experienced palliative care and end-of-life support stress [18].

They stated that their characteristics such as long-term nursing care to patients with CJD, interaction with patients’ relatives, and experience helped them cope with difficulties. The nurses mentioned that the realization of nursing care for CJD in line with scientific and humanistic values was important in making the profession visible to families and other team members. In parallel with this finding, caregivers stated that healthcare professionals’ support was the most important source of support in clinical care [18]. However, the nurses in the present study emphasized that individual differences may pose a threat because it may cause differences in practice; therefore, knowledge and experience should be increased. Thus, in parallel with this result, this study aimed to increase the knowledge of nurses and at the same time, enable them to transfer their experiences by addressing the care of individuals diagnosed with CJD in line with the Model of Living.

### Limitations

The findings regarding the qualitative interview of the nurses who provided care to at least one of the three patients with CJD are limited to the opinions of the nurses participants. The limited sample size due to the rarity of cases diagnosed with CJD, reflecting the experiences of nurses who are responsible for the care of patients and it is difficult for them to reflect their experiences. In addition, we did not determine the duration/level of education and follow current developments in the field of neurology that may affect their care experiences. Nonetheless, this research is one of the first studies to examine in detail the care experiences of nurses who cared for three patients with rare CJD throughout the entire process (except H9).

## Conclusion

This study, which comprehensively examined the nursing care for individuals with CJD in line with the Model of Living and the experiences of nurses during such care. These results revealed that the biggest difficulties for nurses caring for patients with CJD is the fear of contamination. Other difficulties are the inability to answer relatives’ questions due to the fear of contamination and the uncertainty in the prognosis of the disease. This study highlights guide the areas that should be focused on in future nursing care studies and define how nursing care should be for people with CJD, which is a rare and complex disease.

### Implications and recommendations for practice

Recommendations for clinical practice were addressed under five headings: practitioner, educator, researcher, manager and professional in line with the roles of the nurse. In line with the nurse opinions obtained through SWOT analysis from the qualitative part of this research; supporting the strengths of nursing care (within the scope of practitioner role), eliminating weaknesses (within the scope of educator and researcher role), increasing opportunities (within the scope of manager role and being aware of threats (within the scope of professional role) was important. In line with their role as practitioners, nurses should provide nursing care in line with scientific and humanistic values. The implementation of the practitioner role is important in terms of making the profession visible to families and other team members of CJD. Therefore, research should be conducted to increase knowledge and experience. This study results can guide the knowledge of nurses and their training of patients’ relatives. At the same time, it may be recommended that nurse managers provide an environment where nurses can share their concerns and experiences, meet their emotional needs and contribute positively to nursing care through peer support mechanisms and psychosocial support programs.

## Electronic supplementary material

Below is the link to the electronic supplementary material.

Supplementary Material 1

## Data Availability

The data that support the findings of this study are available on request from the corresponding author. The data are not publicly available due to privacy or ethical restrictions.
