# Supplementary material for: Nursing care for individuals with Creutzfeldt–Jakob disease from the perspective of a nursing model and nurses: a case study
Source: BMC Nurs. 2025 Jul 1;24:685. doi: 10.1186/s12912-025-03476-0 (PMC12211252; doi:10.1186/s12912-025-03476-0)
Supplement: Supplementary file 1 — Supplementary Material 1 [file 12912_2025_3476_MOESM1_ESM.docx]

**Supplementary 1 -SEMI-STRUCTURED INTERVIEW FORM**

Hello and thank you for participating in the interview.

We would like to remind you that the interview data will be recorded on a voice recorder and the data will be stored with sensitivity. The voice recorder will be specific to this study and will not be used for other studies and purposes. We would like to state that the answers you, dear participants, give to the questions will be used only for this study and anonymity regarding your identities will be ensured.

You can stop participating at any stage of the study.

**Introductory Information**

1. Nickname:

2. Age:

3. Your marital status and number of children if you are married:

4. Years working as a nurse:

5. Years working in the neurology clinic:

6. Years working in this unit:

7. Your experience working with individuals diagnosed with Creutzfeldt-Jacob (three cases in the clinic):

**Research Questions**

**Question 1.** Could you tell us about your experience of caring for individuals diagnosed with Creutzfeldt-Jacob?

**Question 2.** Could you tell us about the difficulties you perceive in care in line with the cases/facts related to your experience of caring for individuals diagnosed with Creutzfeldt-Jacob?

**Question 3**. What are the difficulties you perceive in caring for individuals diagnosed with Creutzfeldt-Jacob in line with the case/facts related to your experience of caring for individuals diagnosed with Creutzfeldt-Jacob?

**Question 4.** What are the facilitators you perceive in care in line with the case/facts related to your experience of caring for individuals diagnosed with Creutzfeldt-Jacob?

**Question 5.** What are the barriers you perceive in care in line with the case/facts related to your experience of caring for individuals diagnosed with Creutzfeldt-Jacob?

**Question 6.** Your opinions/thoughts/suggestions that you would like to add or mention about the experience of caring for individuals diagnosed with Creutzfeldt-Jacob ..etc.
